# Influence of the application of irrigated water-soluble calcium fertilizer on wine grape properties

**DOI:** 10.1371/journal.pone.0222104

**Published:** 2019-09-05

**Authors:** Rui Wang, Yanbing Qi, Juan Wu, Manoj K. Shukla, Quan Sun

**Affiliations:** 1 School of Agricultural, Ningxia University, Yinchuan, Ningxia, P. R. China; 2 College of Natural Resources and Environment, Northwest A&F University, Yangling Shaanxi, P. R. China; 3 Department of Plant and Environmental Science, College of Agricultural, Consumer and Environmental Sciences, New Mexico State University, Las Cruces, NM, United States of America; State University of Ponta Grossa, BRAZIL

## Abstract

The eastern foot of Helan mountain is a famous production area of high-end wine grapes in China. Excessive application of NPK fertilizer induced deficiency in trace elements, such as calcium, and seriously affected the properties of wine grapes. A vineyard in the eastern foot of Helan mountain was selected to investigate the influence of five different concentration treatments of 15 (T_1_), 30 (T_2_), 45 (T_3_), 60 (T_4_), and 75 (T_5_) kg·ha^-1^ of water-soluble calcium fertilizer (Ca(NO_3_)_2_·4H_2_O) application on grape calcium content, yield and fruit properties. The application of calcium fertilizer significantly increased the calcium content in leaves but reduced that in stems and fruits. The highest grape production of 6560.83 kg·ha^-1^ was achieved at T_5_ calcium fertilizer application, corresponding to increase of 30.92% than that for the control (CK, normal fertilization) treatment. The minimum titratable acid of 4.63 g·L^-1^ in grapes was detected at T_2_ calcium fertilizer application, which was 16.38% lower than CK, however, 13.40% increase in sugar-to-acid ratio was observed at T_2_. At 45 kg·ha^-1^ calcium fertilizer concentration, the anthocyanins content was 6.47 mg·L^-1^, indicating an increase of 53.23% than CK. This study showed that the optimal calcium fertilizer concentration was 30 kg·ha^-1^ with the lowest °Brix, titratable acidity, anthocyanins, the highest total phenols, reducing sugar, sugar-to-acid ratio, and an acceptable concentration of the soluble sugar and tannins.

## Introduction

Terroir of wine refers to the combination of environmental factors including soil, climate, topography [1–6], as well as farming practices [7–8] and gives wine grapes their distinctive character. All these are complex and comprehensive processes and influence grapes properties significantly. With the rapid increase in wine consumption and the increasing emphasis on wine terroir, various measures, such as fertilization, irrigation, and cultivation techniques are widely explored to improve the quality of wine grapes [9–10]. Among these measures, fertilizer application is a straight forward and rapid measure to improve the yield and property of wine grapes [11]. The normal growth of wine grapes depends not only on macronutrients but also on medium and trace elements [12]. High-end wine grapes are usually grown on coarse soil with poor fertility and mineral nutrient deficiency [13]. Thus, investing a large amount of NPK fertilizer to artificially increase economic output leads to the deficiency of trace elements, such as Ca, Mg, Fe, Zn, and B. This severely inhibit the improvement of wine grapes to achieve high yield and quality. Only small amounts of medium and trace elements are needed for grape growth, but they significantly influence grape properties. Therefore, the influence of regional trace elements on wine grape properties should be investigated to improve wine quality, create the terroir of regional wine, and guide the regional characteristic industry.

Calcium, as a component of plant cell walls and intercellular layers, is essential in plants and affects tissue mechanical strength and tolerance of biotic and abiotic stresses. It is an essential mineral nutrient for growth and development of fruit trees [14–16]. However, calcium is a phloem-immobile nutrient in the plant because of easy precipitation. The calcium absorbed by the roots from the soil flows into fruits through the phloem, and could be intercepted by root, -stem, leaf, and fruit during the flow. Therefore, only a small proportion of calcium can flow into fruits, and calcium accumulation in the fruit only occurs during the early growth stage. The frequent appearance of calcium deficiency symptoms has aroused emphasis on extra calcium application during fruit tree growth in recent years [17–18]. Calcium mainly promotes the growth of plant roots and leaves, thereby balancing the physiological characteristics of plants by controlling external media, enhancing fruit flavor, and increasing disease resistance capability [19]. Several studies have shown that calcium fertilizer application increases the yield of cherries, pears, and mangoes and reduce titratable acid content, thereby increasing soluble sugar and vitamin C contents [17, 20–21].

The influence of calcium fertilizer application on the wine and grape properties showed that the application of multiple elements (calcium, magnesium, iron, manganese, copper, zine and boron) fertilizer can increase not only the yield of wine grapes but also the content of tannins, total phenols, and anthocyanins, thereby improving the terroir of wine [22]. Calcium fertilizer application in soil can decrease the rate of dehiscent fruit in Red Globe grapes [23]. The moderate spraying application of calcium fertilizer remarkably contributes to the improvement of the quality of Centennial seedless table grapes by considerably increasing vitamin C and titratable acid contents and fruit firmness, while reducing soluble solid content [24]. Calcium application can also prolong grape storage time after harvest [25–26]. These studies have laid the foundation for the further research of calcium-based micro-fertilizers to enhance fruit tree yield and quality. However, current studies mainly focus on calcium fertilizer application by spreading and spraying. With the development of water and fertilizer integration technology, the influence of water and fertilizer drip irrigation on grape yield and quality remains to be further studied.

Some research have reported climate, terrain and soil at the eastern foot of Helan mountain to be one of the rare production areas for high-quality wines worldwide [13, 22]. While micronutrient deficiency symptom, especially calcium deficiency has been investigated by the researchers because of the excessive NPK fertilizer application with no attendant improvement in the quality of wine [22]. This study was conducted in a vineyard situated at the eastern foot of Helan Mountain, which has the most widely cultivated 4-year-old *Cabernet Sauvignon* as the reference of grape quality in the area. The water-soluble calcium fertilizer (Ca(NO_3_)_2_·4H_2_O), with five different concentration treatments were applied by drip irrigation. The objectives of this research were to: (1) investigate influences of calcium fertilizer on grape calcium accumulation in organs, growth and yield, and grape properties, and (2) recommend the optimal calcium fertilizer concentration application.

## Materials and methods

### Ethics statement

All the sample sites (N38°11′ to 38° 12′ and E 105°55′ to 105° 56) were distributed in the Lilan Winery in Minning Town, Yongning County, Ningxia Province, and permission were granted by the land owner in each site. The field studies did not involve endangered or protected species because all the sample sites were in the vineyard.

### Study area

The eastern foot of Helan Mountain in Ningxia is located between the 37°43′N and 39°23′N and 105°45′E and 106°47′E. The area is in the mild temperate semi-arid climate zone. This area is the alluvial fan in front of Helan Mountain located in the transition zone of Helan Mountain and Yinchuan Basin. Helan mountain is approximately 200 km long from north to south with a total area of approximately 200 000 ha. It is tilted from the southwest to the northeast, and the ground is relatively flat, with an altitude of 1100–1120m. The average annual temperature ranges from 8.6°C to 10°C, the effective accumulated temperature from 3135°C to 3272°C, diurnal temperature from 10°Cto 15°C, and annual precipitation from 180 to 200mm. The annual sunshine duration is 3032h with the sunshine rate of 67%.

The soils of this area are mainly ustic cambosols with aeolian sandy texture. While some soils contain gravels, which are suitable for the growth of wine grapes. The sand, silt, clay, and gravel contents are 85%–90%, 5%–8%, 6%–10%, and about 10%, respectively. The sandy loam soil texture has high-drainage permeability and soil temperature regulation ability, which is conducive to the growth of wine grapes. As shown in Table 1, soil in the vineyard has low nutrient contents with the average SOM content of about 10 g·kg^−1^.The contents of available N, P, and K are also low. Therefore, large amounts of NPK fertilizer are applied to achieve higher wine grape yield.

**Table 1:** Soil properties of vineyard in the eastern foot of Helan mountain.

Depths /cm	pH	Bulk density /g·cm^-3^	Porosity %	SOM /g·kg^-1^	TN / g·kg^-1^	TP / g·kg^-1^	TK / g·kg^-1^	Av-N / mg·kg^-1^	Av-P / mg·kg^-1^	Av-K / mg·kg^-1^
0–20	8.32	1.66	37.41	10.35	0.50	0.29	23.30	42.47	17.73	163.33
20–40	8.47	1.45	45.42	10.21	0.42	0.24	22.27	10.97	4.46	193.33
40–60	8.40	1.37	48.15	10.14	0.17	0.20	20.20	2.80	1.03	80.00

Note: SOM is soil organic matter, TN is total nitrogen, TP is total phosphorus, TK is total potassium, Av-N is available nitrogen, Av-P is available phosphorus, and Av-K is available potassium.

### Experimental design

The experiment was conducted in the vineyards of Lilan Winery with an area of 5.5 ha from April to October 2017. In this study, the water-soluble calcium fertilizer (Ca(NO_3_)_2_·4H_2_O) with calcium oxide (CaO) content of 23.17% was applied. Five concentration treatments applied were 15 (T_1_), 30 (T_2_), 45 (T_3_), 60 (T_4_), and 75 kg·ha^-1^ (T_5_). The calcium fertigated water was applied via drip irrigation at the swelling (July 5) and veraison (August 15) stages at the rate of 3000 m^3^·ha^-1^ in each treatment.

Research area has very cold winter, therefore, grape wines were covered with soil to avoid damage due to the cold air and uncovered during spring. 120 kg·ha^-1^ of urea (with 55.2 kg·ha^-1^ N) was applied at the end of April, and 225 kg·ha^-1^ of urea and diammonium phosphate (N: 40.5 kg·ha^-1^; P_2_O_5_: 108 kg·ha^-1^) were applied after the flowering in late May. Nitrogen, phosphorus, and potassium ternary compound fertilizer was applied at 375 kg·ha^-1^ (N: 56.3 kg·ha^-1^; P_2_O_5_: 56.3 kg·ha^-1^; K_2_O: 56.3 kg·ha^-1^) during the second half of June, and additional fertilizer of top dressing potassium sulfate was applied at 450 kg·ha^-1^ (K_2_O: 225 kg·ha^-1^) at the end of August. Vines without calcium fertilizer application served as the control (CK). The study was conducted using a completely randomized block design, with 3 replications for each treatment. Each experimental plot had an area of 30 m × 50 m, and at least 3 m buffer space was kept between plots. All plots were planted with four-year-old *Cabernet Sauvignon* in north south direction. The vines in the same line were tilted in the same direction when bound to the shelves, with the row spacing of 0.8 m×3 m.

### Grape properties measurement

Before harvest on October 2017, 10 representative clusters of the same part of grapes in each experimental plot were randomly collected and washed with distilled water and stored in the refrigerator at 4°C for later analyses. The leaf, stem and root samples were collected consistently from the same tree. Plant samples were dried and ground, and the calcium content of leaf, stem, root and fruit were measured using atomic absorption spectrophotometer (AAS8000, Skyray instrument company) by wet ashing method (digested with HNO_3_:H_2_SO_4_ = 4:1) [24]. After squeezed of the frozen grape fruit, Brix was determined using a Brix refractometer (PR32 Atago Co. Ltd., Japan). The reducing sugar content was measured using titration with Fehling reagent, and titratable acidity was determined using standardized 0.1 N NaOH (end-point pH 8.2). The soluble sugar content was determined by the anthrone method [27]. The total phenols content was determined by Folin–ciocalteu method using a gallic acid meter [28]. The tannin content was determined by Fehling reagent method using a tannic acid meter [29]. The anthocyanins content was determined by pH differential method using a malvidin-3-glucosinometer [30].

Before grapes were harvested, average berry diameter was calculated by randomly measuring the diameter of 30 grape fruits and average cluster length was calculated by randomly measuring the length of 10 clusters use ruler in the field in each treatment. All the grapes were collected from randomly selected 9 grape trees in each treatment. The average hundred-berry weight was measured and average yield per vine was determined. The theoretical total yield was calculated by multiplying number of trees in each treatment with yield per vine.

### Data analysis

Statistical analysis was performed using Microsoft Excel 2013, and Duncan’s multiple range was used to carry out the significance test on the calcium content of plant organs, as well as morphological and chemical properties of grapes among the treatments. The correlation analysis among morphological properties was performed using the SPSS software.

## Results

### Calcium content in wine grapes organs

**Fig 1:**
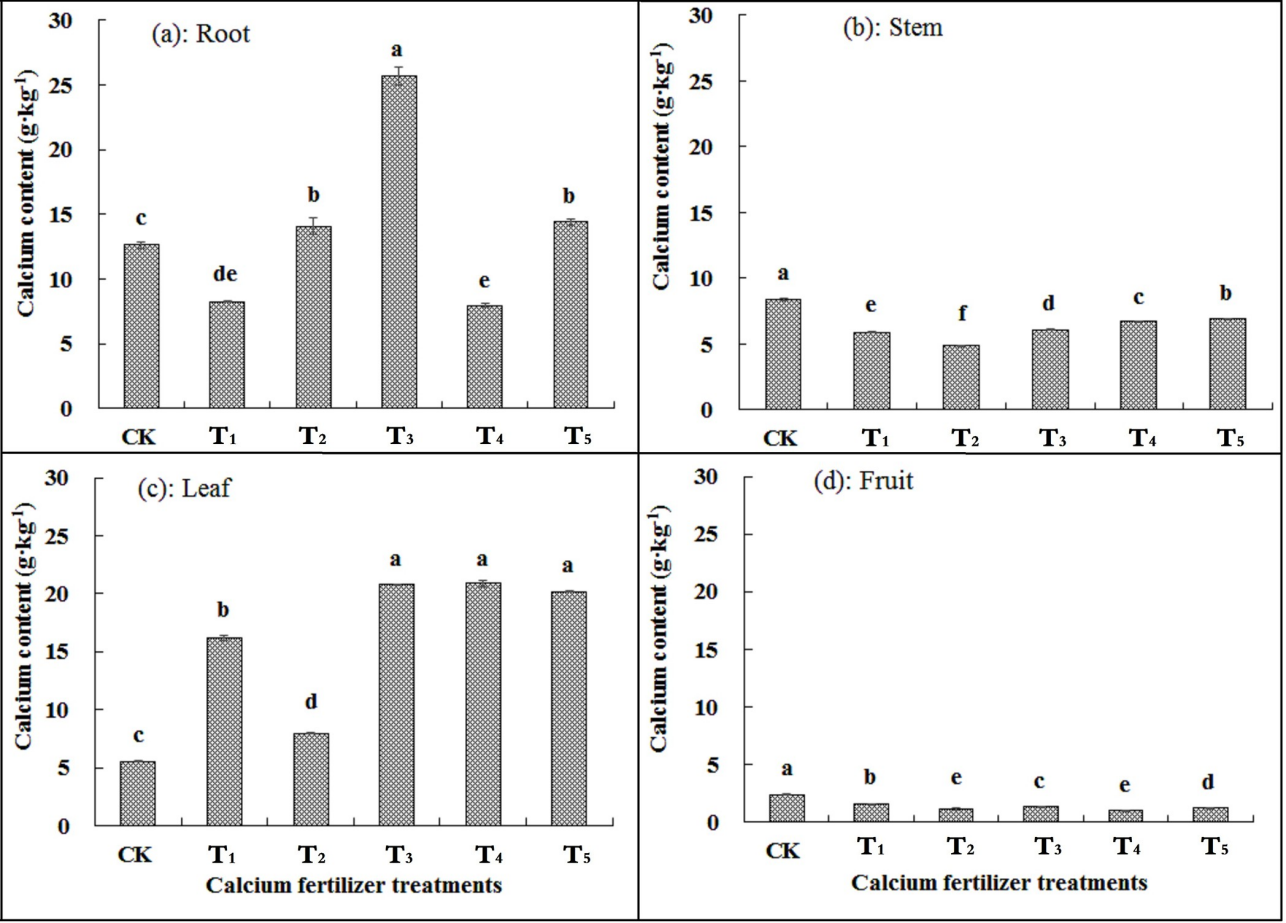
Changes in calcium content in different organs of wine grapes with different calcium fertilizer concentrations. CK means control that unsupplied with calcium fertilizer. Calcium fertilizer treatments from T_1_ to T_5_ corresponded to 15, 30, 45, 60, and 75 kg·ha^-1^ of Ca(NO_3_)_2_·4H_2_O content. Error bars on the volumes means standard deviation.

Fig 1 indicated that calcium accumulation differed among grape organs as well as calcium fertilizer treatments. Calcium content in grape root gradually increased from T_1_ to T_3_ concentrations (Fig 1(A)). Compared with CK, the calcium content in grape root was lower in T_1_ and T_4_ concentrations and higher in other concentrations. Fig 1(B) shows that the application of calcium fertilizer significantly reduced the calcium content in the stem of the grape compared with that in CK, which was reduced by up to 41.90% in the T_2_ concentration and by at least 17.86% at T_5_. The calcium content of the grape leaves were always higher than that for CK (Fig 1(C)). At T_1_ and T_2_ concentrations, the mean calcium content of leaf was 16.21 g·kg^-1^ and 7.96 g·kg^-1^, which responded to 193% and 44% higher compared with the CK, respectively. Increase in leaf calcium content was much higher for T_3_, T_4_ and T_5_ concentrations. Similar to the stem, calcium content in fruit for CK was much higher than those for the fertilizer treatments. In general, application of calcium fertilizer decreased the calcium content by 36.04% to 58.05%, compared with the CK in the grape fruits in all the treatments.

### Morphological indexes and yield of wine grape

Table 2 shows that the application of water-soluble calcium fertilizer via root irrigation affected the morphological indexes and yield of wine grapes, but the effects varied with different concentrations. At T_1_ calcium fertilizer concentration, the hundred-berry weight of the grape was 33% higher than that of CK. For calcium concentration of T_2_ and T_3_, no significant differences were found of the hundred-berry compared with CK. At T_4_, the hundred-berry weight increased to 204.93 g and was 63% higher than that of CK. The effect of calcium application on the berry diameter of grapes was consistent with the hundred-berry weight. The grape berry diameter significantly increased at T_1_ and T_4_ concentrations compared with that of CK, but no significant change was observed at other concentrations.

**Table 2:** Influence of calcium fertilizer treatments on morphological and yield of wine grape.

Treatment	Hundred-berry weight /g	Berry diameter /mm	Cluster length /cm	Yield per vine /kg	Yield /kg·ha^-1^
CK	125.66±4.32 c	11.65±0.32 b	18.93±0.71 a	0.88±0.01 d	5019.00±52.30 d
T_1_	167.09±2.77 b	14.21±0.68 a	13.83±0.95 bc	0.96±0.01 c	5533.69±70.77 c
T_2_	132.28±3.05 c	11.96±0.47 b	11.97±0.41 c	1.07±0.02 b	6099.57±106.58 b
T_3_	128.92±1.69 c	11.15±0.53 b	12.70±1.36 c	0.81±0.00 e	4615.73±6.22 e
T_4_	204.93±3.01 a	13.66±0.16 a	15.87±0.47 b	1.07±0.02 b	6102.38±100.89 b
T_5_	130.56±2.41 c	10.57±0.28 b	13.67±0.74 bc	1.15±0.02 a	6560.83±87.5 a

Note: Significant interactions were at an α = 5%. Different letters in each column indicate significant differences.

As shown in Table 2, calcium fertilizer application significantly shortened the length of the grape cluster. Compared with the CK, all the calcium fertilizer concentration treatments saw a decrease in grape cluster length ranging from 3.06 to 6.96 cm. The shortest and longest cluster length of 11.97cm and 15.87 cm were observed at T_2_ and T_4_ concentrations, respectively. The total yield of grapes and berry diameter was higher than that of CK in all the calcium fertilizer concentrations except at T_3_, although the application of calcium fertilizer shortened the cluster length. Table 2 shows that the yield at T_1_, T_2_, T_4_, and T_5_ significantly higher than that of CK, and the highest yield was achieved at T_5_ by 30.72% increment than CK. At T_2_ and T_4_ concentrations, the yield increased by about 22% than CK. The yield at T_3_ concentration was about 8% lower compared with that of CK.

Among grape morphological indexes, the correlation between berry diameter and hundred-berry weight was significant (p<0.05). Correlations among the remaining indicators was not significant (Table 3). This finding indicated that the application of water-soluble calcium fertilizer had a complicated effect on the morphological indexes during the growth of wine grapes, which did not show a definite pattern with increasing calcium fertilizer concentration.

**Table 3:** Correlation among indicators of grape morphological and yield.

Indicators	Hundred-berry weight	Berry diameter	Cluster length	Yield
Hundred-berry weight	1.000			
Berry diameter	0.818*	1.000		
Cluster length	0.136	0.113	1.000	
Yield	0.295	0.051	-0.227	1.000

Note:

* p<0.05

### Properties of wine grapes

Table 4 shows that the application of water-soluble calcium fertilizer could significantly reduce the total phenols and soluble solid contents in wine grapes. The lowest total phenols content was detected at T_4_ concentration and was 63.95% lower than that of CK. At T_1_ and T_5_ concentrations were about 30% lower compared with CK. While in T_2_, the total phenols were not significantly different than CK. The °Brix of the grapes decreased about 14% at T_1_ and T_5_ concentrations, about 6% at T_2_ and T_4_, and about 10% at T3, compared with the CK.

**Table 4:** Influence of calcium fertilizer treatments on grape properties.

Treatments	Total phenols /mg·g^-1^	Soluble solids /°Brix	Titratable acidity /g·L^-1^	Reducing sugar /%	Reducing sugar/ Titratable acidity	Anthocyanins /mg·L^-1^	Soluble sugar /%	Tannins /g·kg^-1^
CK	3.75 a	27.53 a	5.53 b	20.60 a	37.25 b	4.22 d	16.72 a	16.04 b
T_1_	2.63 b	25.77 b	5.59 b	18.64 c	33.35 c	4.97 c	16.44 a	11.93 cd
T_2_	3.03 ab	23.77 d	4.63 d	19.56 b	42.24 a	2.40 f	12.94 b	17.82 a
T_3_	2.01 bc	24.70 c	4.88 c	16.90 e	34.63 c	6.47 a	11.24 c	15.82 b
T_4_	1.35 c	23.83 d	5.00 c	18.06 d	36.12 b	5.97 b	10.36 d	12.82 c
T_5_	2.60 b	25.80 b	6.50 a	18.87 c	29.03 d	3.10 e	8.36 e	18.60 a

Note: Significant interactions were at an α = 5%. Different letters in each column indicate significant differences.

Table 4 shows that the effects of different concentrations of calcium fertilizer on titratable acid significantly varied. The titratable acidity was increased significantly at T_5_ concentration by 1 g·L^-1^ compared with that of CK, conversely it was decreased significantly at T_2_, T_3_ and T_4_ concentrations, especially at T_2_ concentration, it was decreased by 0.9 g·L^-1^ compared with that of CK. Compared with CK, all the calcium fertilizer treatments decreased the reducing and soluble sugar significantly. The lowest reducing sugar was fond in T_3_ concentration by reduction of 18% compared with CK. At T2 concentration, the reducing sugar slightly decreased by 5%, and it decreased about 13% at T_1_, T_4_ and T_5_ concentrations compared with CK. The soluble sugar content significantly decreased as the calcium fertilizer concentration increased. No significant difference of soluble sugar content was observed at T_1_ concentration, while it was decreased by 50% at T_5_ concentration compared with CK. Table 4 showed that all the calcium fertilizer concentration treatments significant decreased the sugar-to-acid ratio, except at T_2_ concentration by significant increased by 4.99 compared with CK.

As shown in Table 4, the application of water-soluble calcium fertilizer significantly influences the content of anthocyanins and tannins. The anthocyanins content significantly increased at T_1_, T_3_, and T_4_ concentrations, especially at T_3_ concentration, it was increased 53.32% compared with that of CK. While it was significantly decreased at T_2_ and T_5_ concentrations by 43.13% and 26.54% compared with CK, respectively. The tannins content significantly increased at T_2_ and T_5_ concentrations by 11.10% and 15.96% compared with that of CK, respectively. While it was significantly decreased by 25.62% and 20.07% at T_1_ and T_4_ concentrations compared with that of CK, respectively. No significant difference of tannins content was detected between T_3_ concentration and CK.

## Discussion

### Calcium content in wine grape organs

Grape is calciphilous plant, calcium is not only a cell wall component of grape tissue cells but also a major regulator of grape metabolism and development [31]. It has a remarkable impact on the yield and properties of grapes. Therefore, calcium supplementation has been considered as an important measure to increase fruit quality in fruit tree management [32–34]. The results of this study indicated that the application of water-soluble calcium in soil can significantly increase the calcium content of grape leaves. The increase in the leaf calcium content can increases the chlorophyll content of vine leaves and enhances photosynthesis [15], thereby increasing the grape yield. This study showed increased grape yield in all the calcium fertilizer treatments except T_3_ (Table 4). However, the results of this study also showed that the application of calcium fertilizer significantly reduced the calcium content in the stems and fruits of the grapes, this is possibly because calcium is not easily transferred in plants and calcium oxalate blocks the phloem in the fruit stem in the growth anaphase [35]. Similarly, Montanaro et al. [36] reported that a decrease of calcium content in apricot fruit because of the amount of calcium entering the fruits is related to the transpiration rate and the efficiency of the conduit. During the later growth stage, fruit transpiration rate decreased the amount of calcium that entered the fruit [33]. While, most of the researchers have reported calcium application increased the calcium content in the fruits of mango [17], tomato [37] and red globe grape [38], these findings were inconsistent with the results of our study, possibly due to the differences in calcium fertilization method. In our study, calcium fertilizer was applied to the soil via drip irrigation, while calcium in other studies was applied by spraying on plants, and that could have increased uptake by fruits.

### Influence of calcium application on the morphological characteristics and yield of wine grapes

In our experiment, application of calcium fertilizer increased the hundred-berry weight, berry diameter, yield per vine, and total yield in certain calcium concentration (Table 2). These results were consistent with those of Gao *et al*. [39]. The increase of vine morphological characteristics by calcium fertilizer application were due to increased calcium content in the leaves and enhanced photosynthesis to increase the yield. The yield versus calcium treatment responses were similar to Feng and Ling [23]. Feng and Ling reported that the yield increased and reached the maximum when the application of calcium nitrate increased from 0 g to 120 g per red globe grape, however further increase decreased yield.

### Influence of calcium application on the properties of wine grape

The influence of calcium nutrition on grape quality has been extensively investigated in recent years, because it is one of the most important factors that determine fruit quality [40]. Numerous studies have shown that the quality of grapes can be improved through spraying and soil application of calcium fertilizer [8, 37–38, 40–41]. Soluble solids (°Brix) are a reflection of total sugar in wine grapes. The results showed that the application of calcium fertilizer significantly reduced soluble solid content by 6%–14% compared with that of CK (Table 4). These findings were consistent with those of Liu *et al*. [25] on Red Globe grapes, Su and Yang [24] on ‘Centennial seedless’ grapes, and Li *et al*. [17] on mangoes. In contrast, some researchers reported that calcium application increased the content of soluble solids in grapes [23, 41–42], and this observation was inconsistent with the results of this study. This is possible because application of calcium fertilizer significantly decreased the calcium content in fruits (Fig 1), which thereby caused a decrease in the Ca/N ratio as well as a reduction of the utilized Ca^2+^, and weakened sugar metabolism of fruits [43].

Appropriate sugar and acid contents and their ratios are important indicators for determining the maturity of grape fruit and essential factors that affect the properties of wine [44–45]. In this study, the application of calcium fertilizer significantly reduced the titratable acid content in T_2_, T_3_ and T_4_ concentration treatments (Table 4), which was beneficial to improve the quality of wine grapes, and this finding was consistent with the results of other researchers [23,41–42]. While the decreased reducing sugar and soluble sugar were also detected by calcium fertilizer application in this research (Table 4), which will decrease sweetness of the wine. Significant decrease of sugar-to-acid ratio was observed by calcium fertilizer application compared with CK further indicating that the effect of calcium nutrient supplement on the quality of grapes has an optimal range. Liu *et al*. [46] also reported a significant correlation between calcium and sugar-to-acid ratio.

### Recommendation of the optimal calcium fertilizer application

The analysis of the calcium content in organs, morphological characteristics and yield, and properties of vine fruit by setting different calcium fertilizer concentrations indicated that calcium application was beneficial to the improvement of the quality of wine grapes. However, the results also indicated that higher calcium fertilizer application did not improve the vine quality. From the perspective of the morphological characteristics and yield of grapes, the hundred-berry weight, and the total yield increased initially at low concentrations. However, when the concentration was increased to T_3_, these indicators had the tendency to decrease. Therefore, on the basis of morphological characteristics and yield, T_2_ should be recommended as an optimal concentration application. From the perspective of the quality index of wine grapes, the lowest °Brix, titratable acidity, anthocyanins, as well as highest total phenols, reducing sugar, sugar-to-acid ratio, were observed at T_2_ concentration compared with other calcium fertilizer treatments. And at this concentration, the soluble sugar and tannins have an acceptable content. Thus, we recommended the optimal calcium fertilizer concentration was 30 kg·ha^-1^ (T_2_) to achieve high quality vine.

## Conclusions

The use of water-soluble Ca(NO_3_)_2_·4H_2_O is an effective way to increase the yield and quality of wine grapes. Calcium fertilizer application improved the calcium absorption by root and leaf, as well as increased vine yield. The recommended optimal calcium fertilizer concentration was 30 kg·ha^-1^. This calcium fertilizer concentration achieved the lowest °Brix, titratable acidity, anthocyanins, as well as highest total phenols, reducing sugar, sugar-to-acid ratio, and the soluble sugar and tannins have an acceptable content. Therefore, appropriate calcium fertilizer application is recommended to reduce the limitation on wine grape quality due to micronutrient deficiency in the vineyard of the eastern foot of Helan Mountain.
